# Co-limitation of N and P is more prevalent in the Qinghai-Tibetan Plateau grasslands

**DOI:** 10.3389/fpls.2023.1140462

**Published:** 2023-02-15

**Authors:** Kai Dong, Wenjin Li, Yulong Tang, Suhui Ma, Mengluan Jiang

**Affiliations:** ^1^ State Key Laboratory of Herbage Improvement and Grassland Agro-ecosystems, Gannan Grassland Ecosystem National Observation and Research Station, College of Ecology, Lanzhou University, Lanzhou, Gansu, China; ^2^ Institute of Ecology, College of Urban and Environmental Sciences, and Key Laboratory for Earth Surface Processes of the Ministry of Education, Peking University, Beijing, China

**Keywords:** meta-analysis, nutrient limitation, nitrogen, phosphorus, plant biomass and diversity, alpine grassland, Qinghai-Tibetan Plateau

## Abstract

**Introduction:**

Over the past three decades, the view of nutrient limitation has transferred from single-nutrient limitation to multiple-nutrient limitation. On the Qinghai-Tibetan Plateau (QTP), many nitrogen (N) and phosphorus (P) addition experiments have revealed different N- or P-limited patterns at many alpine grassland sites, whereas it is not clear what the general patterns of N and P limitation across the QTP grasslands.

**Methods:**

We performed a meta-analysis, containing 107 publications, to assess how N and P constrained plant biomass and diversity in alpine grasslands across the QTP. We also tested how mean annual precipitation (MAP) and mean annual temperature (MAT) influence N and P limitations.

**Results:**

The findings show that plant biomass in QTP grasslands is co-limited by N and P. Single N limitation is stronger than single P limitation, and the combined positive effect of N and P addition is stronger than that of single nutrient additions. The response of biomass to N fertilization rate shows an increase firstly and then declines, and peaks at approximately 25 g N·m^-2^·year^-1^. MAP promotes the effect of N limitation on plant aboveground biomass and diminishes the effect of N limitation on belowground biomass. Meanwhile, N and P addition generally decline plant diversity. Moreover, the negative response of plant diversity to N and P co-addition is strongest than that of single nutrient additions.

**Discussion:**

Our results highlight that N and P co-limitation is more prevalent than N- or P-limitation alone in alpine grasslands on the QTP. Our findings provide a better understanding of nutrient limitation and management for alpine grasslands on the QTP.

## 1 Introduction

Nutrient limitations, particularly those of nitrogen (N) and phosphorus (P) limitations, have profound influences on plant community biomass and composition (Elser et al., 2007). Typically, plant growth, development, reproduction, and changes in plant productivity and diversity are regulated by N and/or P supplies (Elser et al., 2007; Olde Venterink, 2011; Zhang et al., 2019). Some classical methods, such as nutrients addition experiments, plant stoichiometry and foliar nutrient resorption, have demonstrated that N and P limitations are extensive across terrestrial ecosystems (Elser et al., 2007; LeBauer and Treseder, 2008; Yan et al., 2017; Du et al., 2020). A recent global-scale study predicted that 18% of terrestrial natural lands are N limitation, P limitation accounts for 43% and the rest of 39% are N and P co-limitation area or slightly limited by single nutrient (Du et al., 2020), indicating that P is a more predominant limiting nutrient factor than N globally. The substrate soil-age hypothesis also predicts that nutrient limitation can be changing across the globe (Chadwick et al., 1999). Worldwide mapping of N and P limitation patterns shows that terrestrial ecosystems of boreal and temperate coniferous forests, mountainous grasslands and shrublands, and tundra are prevalently N-limited, while P limitation predominates in (sub-) tropical and temperate areas (Du et al., 2020). Therefore, nutrient limitation is spatially heterogeneous across various ecosystem types.

Spatial heterogeneity and climate factors can influence the magnitude of nutrient limitations. Not surprisingly, N and P limitations demonstrate obviously latitudinal and elevational patterns. Generally, N is more restrictive in high latitude and high altitude zones (i.e., low-temperature areas) (Du et al., 2020), whereas P limitation gradually increases from boreal and Arctic regions to tropical forests in the Northern Hemisphere (Chadwick et al., 1999). This is due to increasing precipitation and temperature could strongly diminish N limitation than P limitation (Du et al., 2020; Hou et al., 2020). Interestingly, there is mounting evidence that plant biomass and diversity are limited by multiple nutrients (i.e., co-limitation) in many terrestrial ecosystems (Olde Venterink, 2011; Fay et al., 2015). Therefore, the concept of nutrient limitation has extended from an earlier perception of single-nutrient limitation to the hypothesis of multiple nutrients co-limitation. The responses of plant biomass and diversity to simultaneously N and P fertilization could be synergistic, additive, and non-additive (Harpole et al., 2011; Olde Venterink, 2011; Fay et al., 2015; Wang et al., 2022a). However, previous studies on N and P limitation patterns are usually at the global scale, and specific knowledge on a local/regional scale (e.g., in alpine grasslands on the Qinghai-Tibetan Plateau) is often not clear.

The Qinghai-Tibetan Plateau (QTP), with a vast elevation gradient ranging from 2000 m to 5000 m, is undergoing huge environmental changes (e.g., global climate change and N deposition) (Piao et al., 2019). The alpine grassland, as the most widely distributed and key terrestrial ecosystems in the QTP (Zhang et al., 2014; Wang et al., 2022b), is an ideal experimental site to examine N and P limitation patterns due to low soil N and P contents resulting from low decomposition and mineralization rate (Ren et al., 2016). In the QTP throughout the past 5 decades, numerous N and P fertilization experiments were conducted. Differently, the alpine grasslands on the QTP appeared N limitation (Ren et al., 2016), P limitation (Yang et al., 2014), or N and P co-limitation (Ren et al., 2010). However, the general patterns of N and P limitation in alpine grasslands across the QTP scales remains unclear.

A clear understanding of general N and P limitation pattern in the QTP grasslands can guide the maintaining and restoration management of plant biomass and diversity of alpine grasslands. Here, we operated a meta-analysis to investigate whether plant biomass and diversity of alpine grasslands on the QTP are limited by N and P. Specifically, we mainly to answer: 1) What are N and P limitation patterns of alpine grasslands on the QTP, and which limitation pattern is more prevalent? 2) How does plant diversity respond to N and P addition across QTP? 3) How do mean annual precipitation (MAP, mm) and mean annual temperature (MAT, °C) influence N and P limitation patterns? We hypothesized that N limitation alone is more prevalent than N and P co-limitation in alpine grasslands because of high altitude zones (Du et al., 2020), and MAT is a key affecting factor for nutrient limitation due to low temperature in this region.

## 2 Materials and methods

### 2.1 Data acquisition

We gathered data from articles through searching in Web of Science (https://apps.webofknowledge.com) and the China National Knowledge Infrastructure (https://www.cnki.net) with the terms [(Qinghai-Tibet Plateau OR Qinghai-Tibet* Plateau) AND (nitrogen addition* OR N addition* OR nitrogen fertili* OR N fertili* OR nitrogen deposit* OR N deposit* OR nitrogen enrich* OR N enrich* OR nitrogen application OR N application OR nitrogen deposit* OR N deposit* OR phosph* addition* OR P addition* OR phosph* fertili* OR P fertili* OR phosph* deposit* OR P deposit* OR phosph* enrich* OR P enrich* OR phosph application OR P application OR fertilization) AND (biodivers* OR divers* OR species rich* OR biomass OR product*) AND (plant* OR grass* OR herb* OR forb* OR sedge* OR legume*)] through to March 1, 2022. We preliminary screened the titles and abstracts of all the publications that were retrieved from the two search platforms. Afterward, we evaluated each article and collected data with the following criteria: 1) Studies was based on fertilization experiments in natural alpine grasslands on the QTP; 2) Studies focused on the impacts of nutrient addition (N and/or P and/or N and P co-addition *vs*. Control) on alpine grasslands; 3) Articles reported at least one of the following variables: vascular plant aboveground biomass (AGB) and belowground biomass (BGB) of total and/or functional groups (grass, sedge, non-leguminous forb, legume) and biodiversity (plant richness, Shannon-Wiener index, Pielou’s evenness index); 4) Studies reported the selected variables of values, including means, standard deviations (SD), or standard error (SE), sample sizes. A total of 107 articles were retained, containing multiple pairwise experiments (i.e., nutrients addition *vs*. control). Details of the articles are listed in **Supplementary Material**.

**Figure 1:**
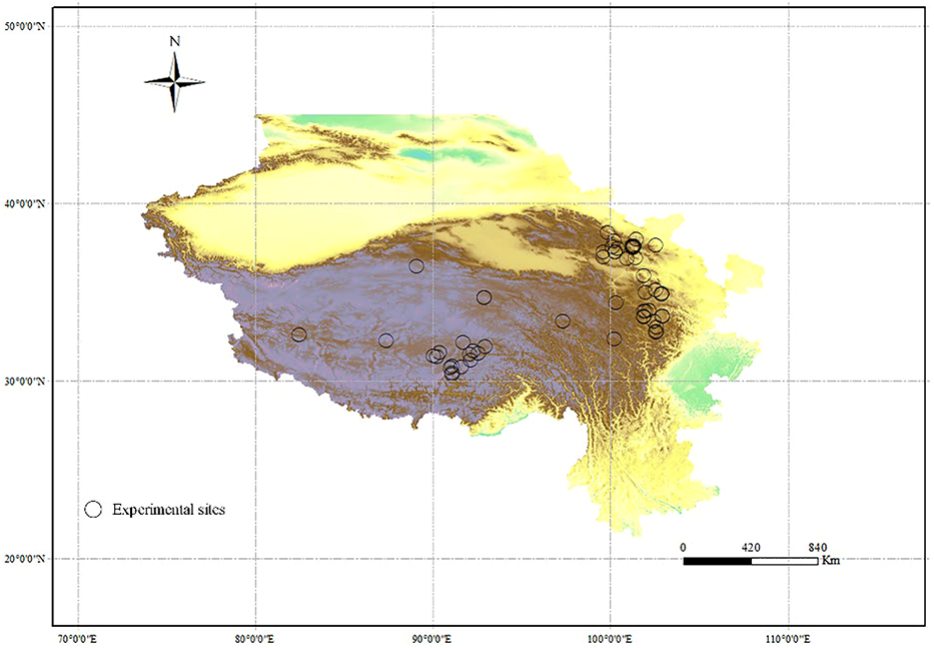
Experimental sites of each article in the meta-analysis in alpine grasslands on the QTP.

We directly gathered data from the tables in the original main text and online data of each article, and used the WebPlotDigitizer (https://automeris.io/WebPlotDigitizer/) to extract the data presented in figures. For each study, we extracted the values of relative variables (mentioned in criteria 3) from the control and nutrient addition treatments. We also recorded citations, year, latitude, longitude, fertilizer type, fertilization rate, and experimental period of each study (**Figure 1**). MAP and MAT were acquired from the original publications, or, if not presented, from the WorldClim database (https://www.worldclim.org/data/index.html). For experiments excluding mammals physically, we retained those that prohibited grazing only in growing season.

### 2.2 Data analyses

We calculated the log response ratio(lnRR) for each data pair (Hedges et al., 1999):


(1) $$lnRR=\ln\left(\frac{{\bar{X}}_{e}}{{\bar{X}}_{c}}\right)=ln{\bar{X}}_{e}-ln{\bar{X}}_{c}$$

where ${\bar{\text{X}}}_{\text{e}}$ and ${\bar{\text{X}}}_{\text{c}}$ respectively represent the arithmetic mean values of each variable in nutrient addition and control manipulations.

We calculated the variance (*v*) of each lnRR as:


(2) $$v=\frac{S_{e}^{2}}{N_{e}{\bar{X}}_{e}^{2}}+\frac{S_{c}^{2}}{N_{c}{\bar{X}}_{c}^{2}}$$

where *S_e_* and *S_c_* represent the standard deviations of means, and *N_e_* and *N_c_* are the sample sizes in nutrient addition and control manipulations, respectively.

We used the escalc() and rma.mv() function from the R package “metafor” (Viechtbauer, 2010) to calculate the weighted effect sizes and 95% confidence intervals (CI), with each study nested in the paper as random effect to account for non-independence of data (i.e., multiple studies in a single paper). We considered the effects size (LnRR) to be significant once the 95% CI of mean value of each variable did not overlap with 0. We tested the overall effects of nutrient addition on plant biomass and diversity. Then, we used meta-regression (Borenstein et al., 2009) from the “metafor” package to test whether the influence of nutrient addition on plant biomass and diversity in alpine grasslands on the QTP depended on MAT and MAP. We estimated the moderator through the QM statistic and corresponding P-value to explain the amount of heterogeneity. Funnel plot asymmetry from Kendall’s rank test was used to assess to assess possible publication bias. Finally, we used linear regression analyses and fitted polynomial curves to explore the threshold of the effects of nutrient addition. All statistical analyses were performed in R platform [version 4.0.3] (R Core Team, 2020).

## 3 Results

### 3.1 Nutrient limitations in alpine grasslands on the QTP

**Figure 2:**
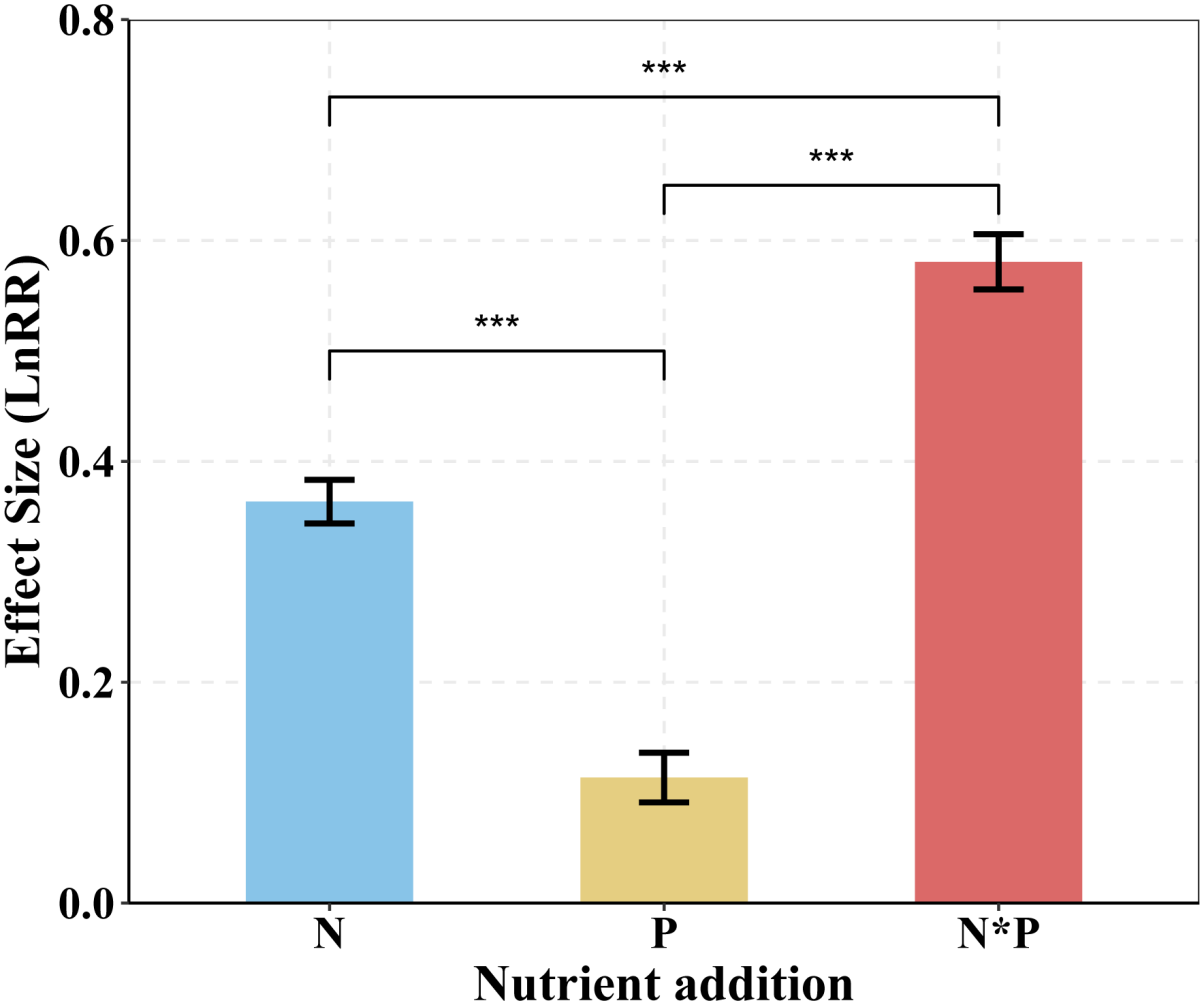
Responses of AGB to single N or P addition or to N*P addition in alpine grassland on the QTP. Mean ± SE are shown. AGB, aboveground biomass. N*P, N and P co-addition. ANOVA was used to analyze the response ratio (LnRR) of plant AGB for each nutrient treatment. The least significant difference (LSD) test was used to separate means at *P*= 0.05. The statistically significant effect denoted by *p < 0.05, **p < 0.01, ***p < 0.001.

**Figure 3:**
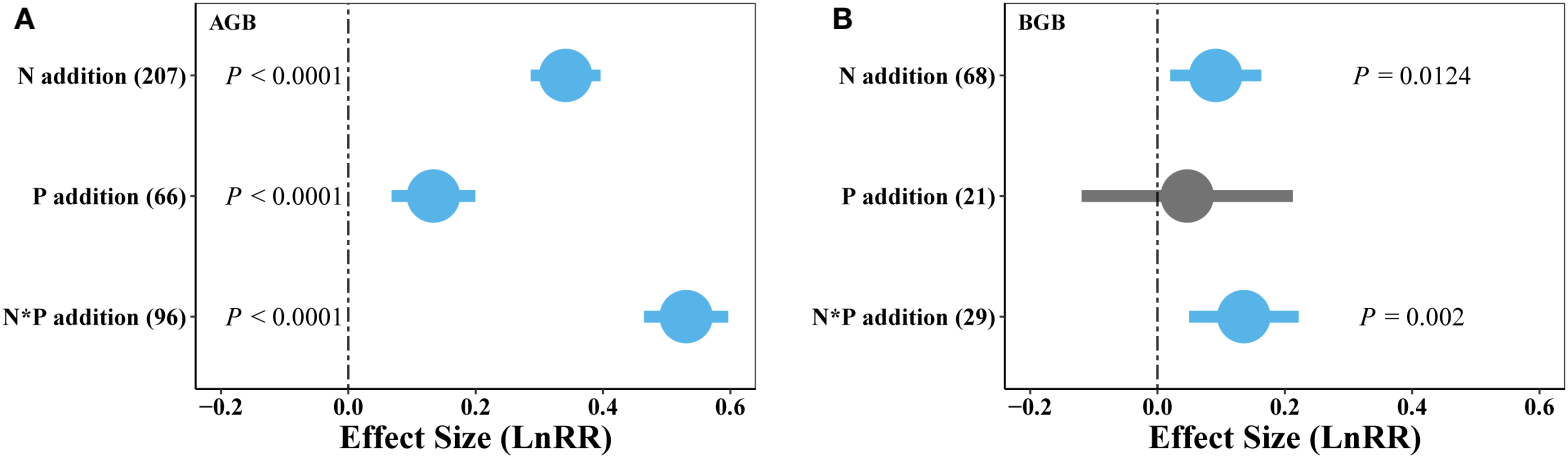
Effects of single N or P, and combined N and P (N*P) addition on weighted response ratio (LnRR) of AGB **(A)** and BGB **(B)** in alpine grassland on the QTP. The numbers in brackets indicate the number of studies for each treatment. Error bars represent 95% confidence intervals. The vertical dashed line represents the weighted effect size = 0. The effect of nutrient addition was considered statistically significant if the 95% CI did not overlap zero. Positive (blue) and neutral (grey) effects are shown.

Our findings showed that the positive effects of N addition on plant aboveground biomass were larger than did P addition, with combined N and P addition showing the strongest positive impacts on plant AGB (**Figures 2**, **3A**). Additionally, BGB significantly increased through single N or combined N and P additions, whereas P addition did not show significant effects on BGB (**Figure 3B**). These results demonstrate a prevalent N and P limitation pattern in alpine grassland on the QTP.

**Figure 4:**
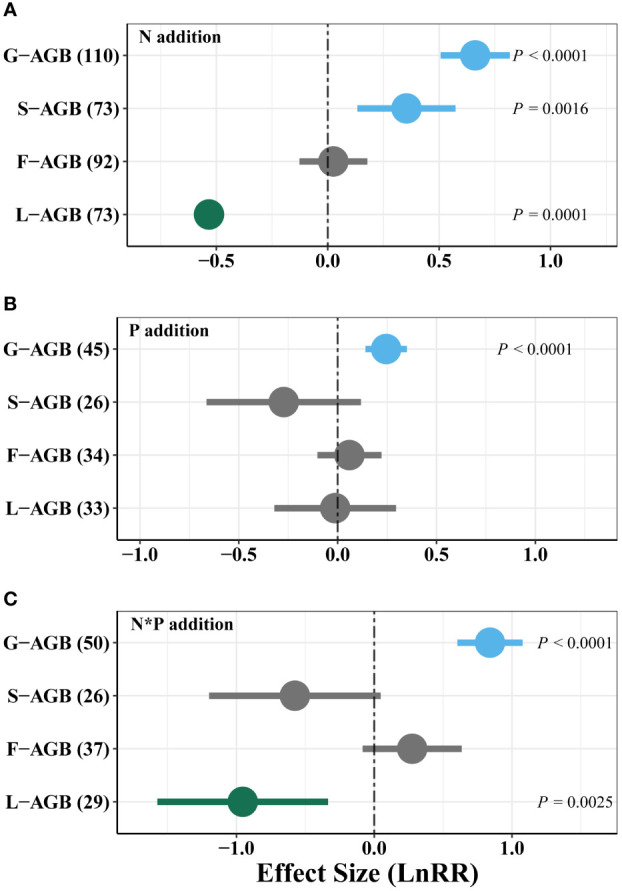
Effects of single N **(A)**, P **(B)**, and combined N and P (N*P, **C**) addition on weighted response ratio (LnRR) of functional groups’ AGB in alpine grassland on the QTP. G-AGB, grass aboveground biomass; S-AGB, sedge aboveground biomass; F-AGB, non-leguminous forb aboveground biomass; L-AGB, legume aboveground biomass. Positive (blue), negative (green) and neutral (grey) effects are shown.

N addition strongly increased AGB of grasses and sedges, but reduced legume AGB (**Figure 4A**). P addition promoted AGB of grasses but had weak effect on other functional groups (**Figure 4B**). Moreover, combined N and P addition significantly increased grass AGB, but decreased legume AGB (**Figure 4C**). The combined N and P addition had a similar effect size with single N addition.

**Figure 5:**
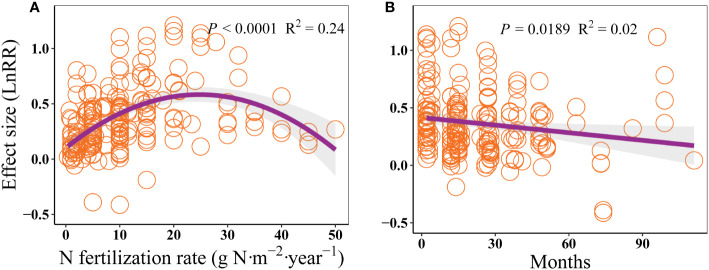
Effects of N fertilization rate (g N·m^-2^·year^-1^, **A**), and experimental period (month, **B**) on the response of AGB in alpine grassland on the QTP.

The effect size of AGB to N addition raised firstly and then declined with increasing N fertilization rate, which peaked at approximately 25 g N·m^-2^·year^-1^ (**Figure 5A**). This pattern was mainly attributed to the response of grasses and sedges, which showed hump-shaped relationships between the effect size of AGB and N fertilization rate (**Figure S1**). Moreover, the effect size of AGB gradually decreased with increasing experimental period (**Figure 5B**).

**Figure 6:**
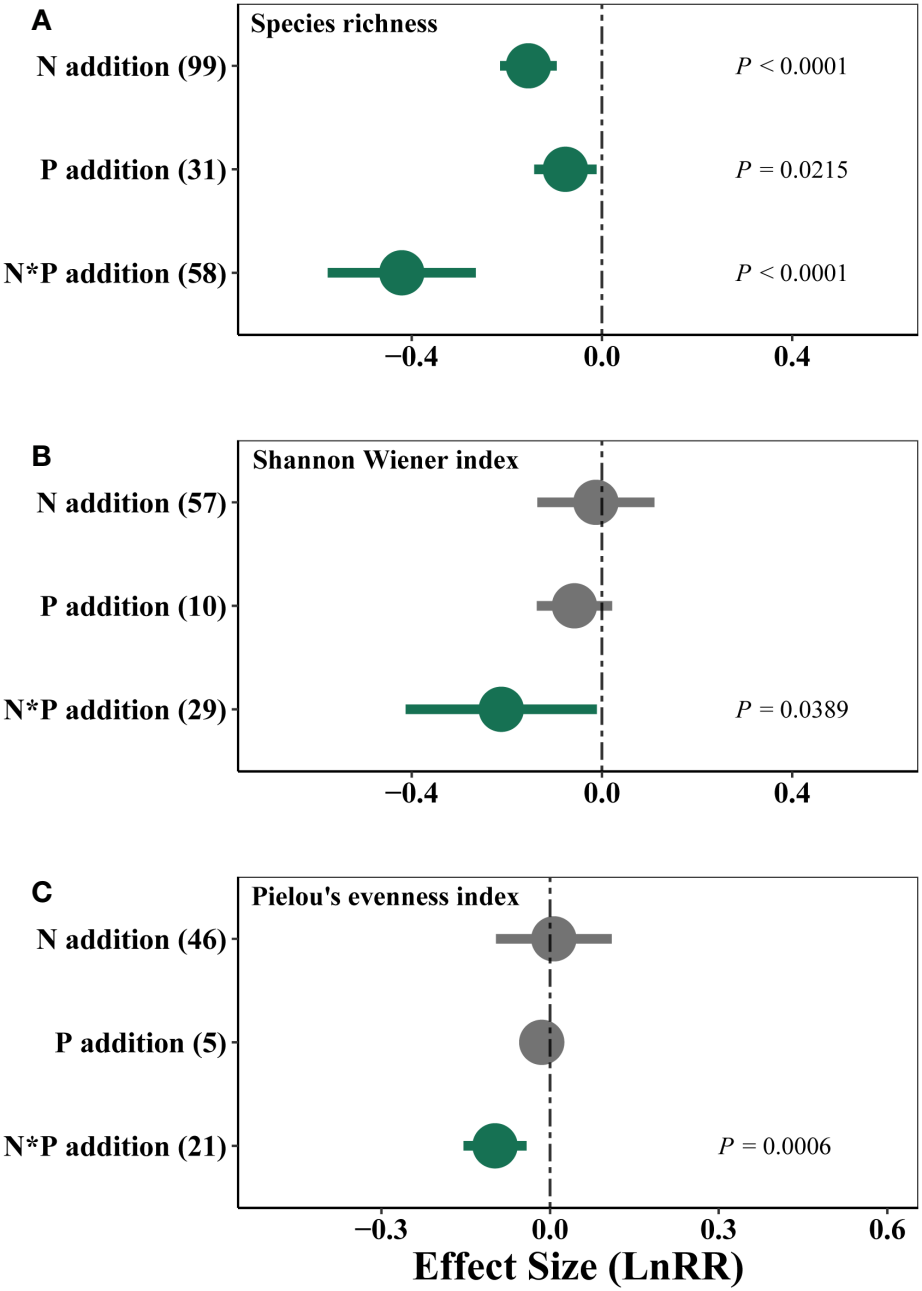
Effects of single N or P, and combined N and P (N*P) additions on weighted response ratio (LnRR) of species richness **(A)**, Shannon Wiener index **(B)** and Pielou’s evenness index **(C)** in alpine grassland on the QTP.

Nutrient addition showed negative effects on species richness, with N and P co-addition showing the strongest negative effects (**Figure 6A**). N and P co-addition also reduced Shannon diversity and Pielou’s evenness of plant communities (**Figures 6B, C**).

### 3.2 Climate mediated nutrient-addition effects on plant biomass

**Figure 7:**
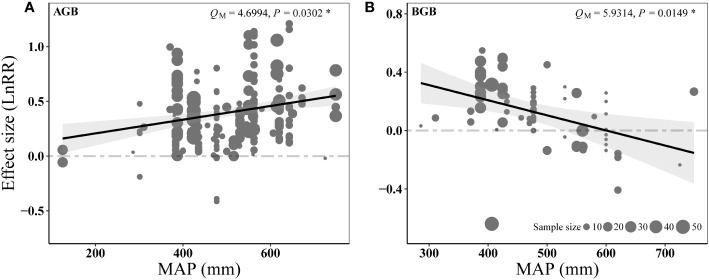
Results of meta-regressions relating the N addition effects on AGB **(A)** and BGB **(B)** (effect size, LnRR) to the MAP. The paralleled gray dashed lines represent an effect size (LnRR) of zero.

The influence of N addition on AGB was positively related to MAP (**Figure 7A**), whereas the effect size of N addition on BGB showed the opposite (**Figure 7B**). The response to P addition did not show significant relationship with precipitation (**Figures S2A, B**). However, increasing MAT slightly decreased the positive effect of P and N addition on aboveground biomass (*P* = 0.068 in **Figure S2C**, *P* = 0.0676 in **Figure S3A**).

## 4 Discussion

We demonstrated that N and P addition/co-addition considerably increased plant aboveground biomass, whereas reduced plant diversity in alpine grasslands on the QTP. AGB of plant responses to N addition are influenced by N fertilization rate and experimental duration. Additionally, we found that MAP, not MAT could change the magnitude of N limitation on plant growth, rather than P limitation or N and P co-limitation. This indicates that alpine grassland on the QTP not only is limited by N or P nutrient alone, more importantly, but also is strongly independent N and P co-limitation (Olde Venterink, 2016). This also supports that remarkable interactive effects of combined N and P enrichment are widespread in terrestrial ecosystems, which can be mediated by climatic conditions, such as MAP (Elser et al., 2007; Fay et al., 2015).

### 4.1 Nitrogen limitation

Single N addition promoted plant AGB and BGB, but decreased species richness, indicating N limitation on alpine grasslands of the QTP. It is well known that N, as an crucial component of chlorophyll and a variety of photosynthetic enzymes, can facilitate plant growth and reproduction (Kattge et al., 2009). Additionally, N supplies can encourage the growth of roots to meet other nutrient requirements (Yang et al., 2009; Peng et al., 2017). N limitation on plant AGB varied with plant functional groups. We found that N fertilization favored to increasing AGB of grasses and sedges, and decreased to the accumulation of legumes’ AGB, but did not change AGB of non-leguminous forbs, which suggests that N limited the growth of grass and sedge. Thus, in alpine grasslands on the QTP, the positive responses of total plant biomass to N addition mainly contribute to the increase in grass and sedge biomass. Contrarily, the response of higher aboveground biomass is accompanied by a decline of species richness through intense resource competition (e.g., nutrients and/or light competition) (Aerts et al., 2003; Olde Venterink, 2011). Due to light competition, presumably self-shading induced by rapid growth of grasses (e.g., *Elymus nutans*) as a result of N addition has a greater negative impact on short stature plants, leading to the loss of species diversity. N addition also might generate the toxic effects, when excessive nutrient addition to natural vegetation exceed the homeostasis regulation capability of plants (Güsewell, 2004). Thus, the productivity of plants declines as N fertilization rate exceeded threshold value. Our findings also demonstrated that the threshold of N fertilization rate is 25 g N·m^-2^·year^-1^, supporting the above-mentioned toxic effects’ prediction.

The impacts of N addition on AGB and species richness at QTP grasslands diminished over experimental duration. A robust explanation is long-term and continuous N addition increased P limitation. This is because N addition significantly reduced P concentration and enhanced plant N:P ratio, but plant rapid growth required more P, thereby mitigating the impacts of single N limitation (Li et al., 2016; Li et al., 2022).

N fixation and mineralization are biological processes that are hugely affected by climatic conditions (Houlton et al., 2008). In QTP, the N limitation of plant biomass was associated with MAP, while MAP had a positive relationship for AGB and negative relationship for BGB. The phenomenon reveals a shift of plant biomass allocation from belowground to aboveground with improved soil N and water condition. This might be due to the fact of that cold climate in alpine grasslands inhibits water evaporation, and the native plant species have evolved mechanisms to adapt to moisture condition (He et al., 2006). Therefore, more N and water allocation will enter the above-ground parts of the plant community. In addition, Eskelinen and Harrison (2015) have proposed the water and N co-limitation hypothesis, where emphasizes the increase of N-fixers caused by water addition. Accordingly, as a result of increased precipitation, enhanced N-fixers may promote decomposable litters, and change soil microorganism communities and nutrient cycling, further, alter the demands for nutrients by plants (Suttle et al., 2007; Eskelinen and Harrison, 2015; Zong et al., 2021). A recent study across the Tibetan permafrost region also suggests that the increased plant N requirement and loss of gaseous N caused by climate warming and wetting could strengthen the effects of N limitation on plant growth (Kou et al., 2020). Contrarily, LeBauer and Treseder (2008) have shown that N limitation is independent of temperature and precipitation in grassland. We did not find strong correlations between the effects of fertilization on plant biomass and MAT due to the prolonged cold temperature and narrow amplitudes in alpine grasslands on the QTP.

In general, N limitation is more severe than P limitation on the QTP since P weathering (a chemical process) is far less sensitive to temperature than N mineralization (a biological process) (Heerwaarden et al., 2003; Ren et al., 2010). Therefore, the low temperature in alpine grasslands on the QTP limits soil mineral nutrients availability, predominantly N availability (KÖener, 2003).

### 4.2 Phosphorus limitation

Although P effect was less than N effect in our meta-analysis, P limitation is also remarkable in alpine grasslands on the QTP. A main reason is that plant growth and reproduction can be limited once the supply of P for photosynthesis machinery (such as rRNA or proteins) is insufficient (Sterner and Elser, 2002). Additionally, we found that the response to P addition was not significantly correlated with MAP and MAT, suggesting that the response to P addition is not sensitive to precipitation and temperature, compared to the response of N in QTP.

Our findings also demonstrated that single P addition promoted AGB rather than BGB, but significantly reduced species richness, presumably because plant competition for nutrients shift into light competition (Borer et al., 2014). Evidently, Li et al. (2022) suggested that P addition significantly raised plant height by 18.8% in grasslands, while Ren et al. (2010) found a reduction in light penetration into the plant canopy. Moreover, soil acidification caused by excessive P addition may also be a reason for the reduction of plant species richness (Borer et al., 2014).

### 4.3 Nitrogen and phosphorus co-limitation

Our synthesis revealed that increase in plant biomass and decrease in plant diversity induced by N and P co-addition were greater than that of single N or P addition, suggesting that N and P co-limitation is more prevalent in alpine grasslands across the QTP. This is consistent with the results of Wang et al. (2020) in degraded grasslands of the QTP, they found that N and P co-addition promoted plant growth, but decreased plant diversity. The decomposition of soil organic matter and the release of nutrients in alpine grasslands on the QTP may be limited by low basic N and P contents as a result of lower temperatures and less rainfall. Harpole et al. (2011) proposed that independent co-limitation is more prevalent in areas with low-level total N and P. Apparently, as limited nutrients, N and P co-addition increase soil N and P contents, thereby enhance the N and P availability. Meanwhile, N and P addition could promote the synthesis of enzymes and mycorrhizal activity, which is advantageous for plants to absorb nutrients (Rowe et al., 2008). Multiple nutrients co-addition alters resource competition and community composition, thus co-limitation directly emerges at the community level (Danger et al., 2008).

Generally, N and P co-addition significantly decreased plant diversity, reflecting the decline of niche dimension due to surging nutrient limitation (Interlandi and Kilham, 2001; Ren et al., 2010). The homogenization of resources caused by nutrient supplies, which diminishes niche differentiation and increases the number of dominant species. In accordance with Tilman’s theory (Tilman, 1982), the dynamics of plant community are driven by resource competition (for limited resources). The general increase in plant biomass caused by nutrients co-addition intensifies resource competition in turn decreases species diversity as a result of competitive exclusion (Grime, 1973; Wang et al., 2022a). We found that N limitation has more negative effects on species richness than P limitation. According to the mechanism of resource pre-emption competition, the superior competitor will receive a resource from rhizosphere faster than its neighboring rivals (Craine et al., 2005). Belowground competition for the acquisition of nutrients might be more critical under N limitation as opposed to P limitation (Olde Venterink, 2011), because N limitation is more obvious in vegetation with a high biomass of roots, strong photosynthetic activity, rapid growth rate and high nitrogen productivity (Gusewell, 2005; Lambers et al., 2008; Lambers et al., 2011; Olde Venterink, 2011). Therefore, magnitude of increase in plant belowground biomass caused by different nutrients addition can reduce plant diversity in alpine grasslands on the QTP.

Studies on alpine grasslands also found that soil available N pools dramatically rose by 24% between the 1980s and 2010s, while available P and K pools declined by 3% and 23%, respectively (Tian et al., 2019), suggesting that Tibetan alpine grasslands could shift from N limitation to P or K-limited in the future (Tian et al., 2019). This was also supported by Zong et al. (2021), who revealed that the pattern of nutrient limitation in alpine grasslands on the QTP shifted from N limited to N and P co-limited, but depending on precipitation. A possible reason is that increasing soil N availability with the increase of N deposition, climate warming and wetting on the QTP may further exacerbate P deficiency (Aerts et al., 1992) and potentially increase other nutrients limitation, such as P, K, or micronutrients (Mahowald et al., 2008). Alternatively, because the uptake of one nutrient is closely correlated with that of other nutrients (Fay et al., 2015), single nutrient addition will rapidly induce limitation by the alternative nutrient (Elser et al., 2007), as a reason of P limitation induced by N addition could be compensated by N and P co-addition. Many studies have also demonstrated the positive effects of N and P co-addition on ANPP are greater than N or P addition alone on the QTP (Ren et al., 2010; Ren et al., 2016; Wang et al., 2022b). Therefore, N and P independent co-limitation is more widespread in alpine grassland on the QTP.

## 5 Conclusion

Our meta-analysis shows that N and P independent co-limitation is a more prevalent pattern than single N or P limitation in alpine grasslands on the Qinghai-Tibetan Plateau. This did not support our hypothesis that N limitation alone is more prevalent. Meanwhile, nutrients addition and MAP have stronger interactive effects on alpine grasslands than interactive effects of nutrients addition and MAT, suggesting that MAP has more important role in mediating nutrient limitation than MAT in this region. N and P co-addition results in a greater reduction of species richness than that of N or P addition alone, reconfirming the resource competition hypothesis and the compensatory effects between limited resources, and it also suggests that excessive fertilization and increased N and P deposition will result in rapid species loss. In summary, our findings will provide a scientific basis for helping to understand nutrients limitation patterns, which will be conductive to fertilization management in degraded alpine grasslands on the QTP under the global climate change.

## Data availability statement

The original contributions presented in the study are included in the article/**Supplementary Material**. Further inquiries can be directed to the corresponding author.

## Author contributions

All authors conceived the project. KD, YT processed data and conducted the meta-analysis. WL, SM and MJ helped with data analyses and manuscript revision. All authors contributed to the article and approved the submitted version.
