# Supplementary material for: Co-limitation of N and P is more prevalent in the Qinghai-Tibetan Plateau grasslands
Source: Front Plant Sci. 2023 Feb 15;14:1140462. doi: 10.3389/fpls.2023.1140462 (PMC9975565; doi:10.3389/fpls.2023.1140462)
Supplement: Supplementary file 1 [file DataSheet_1.docx]

Supplementary material


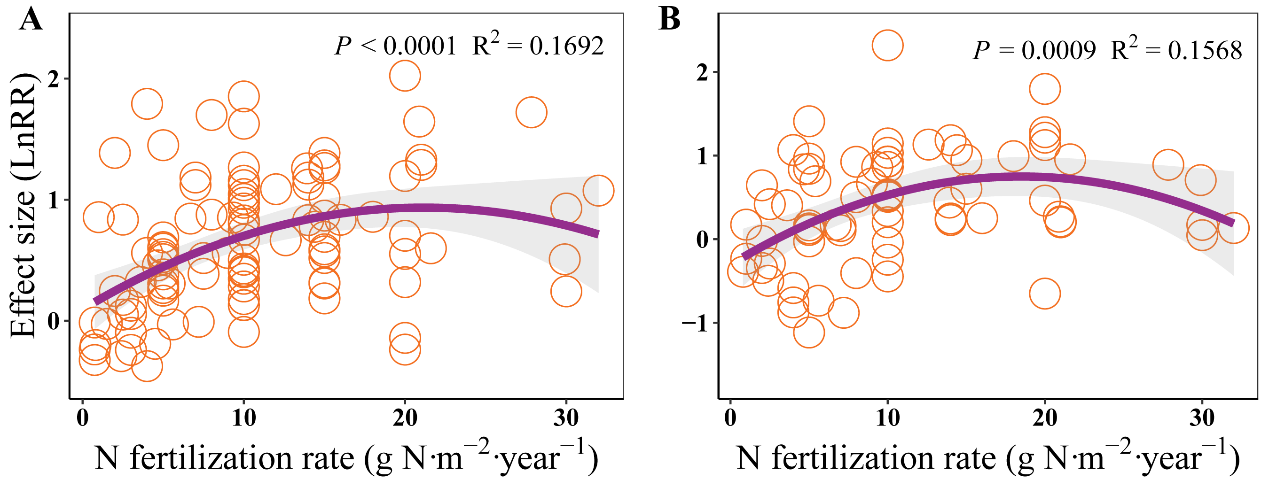


**FIGURE S1.** Effects of N fertilization rate (g N∙m^-2^∙year^-1^) on the response of grass AGB (A) and sedge AGB (B) in grassland of the Qinghai-Tibetan Plateau.


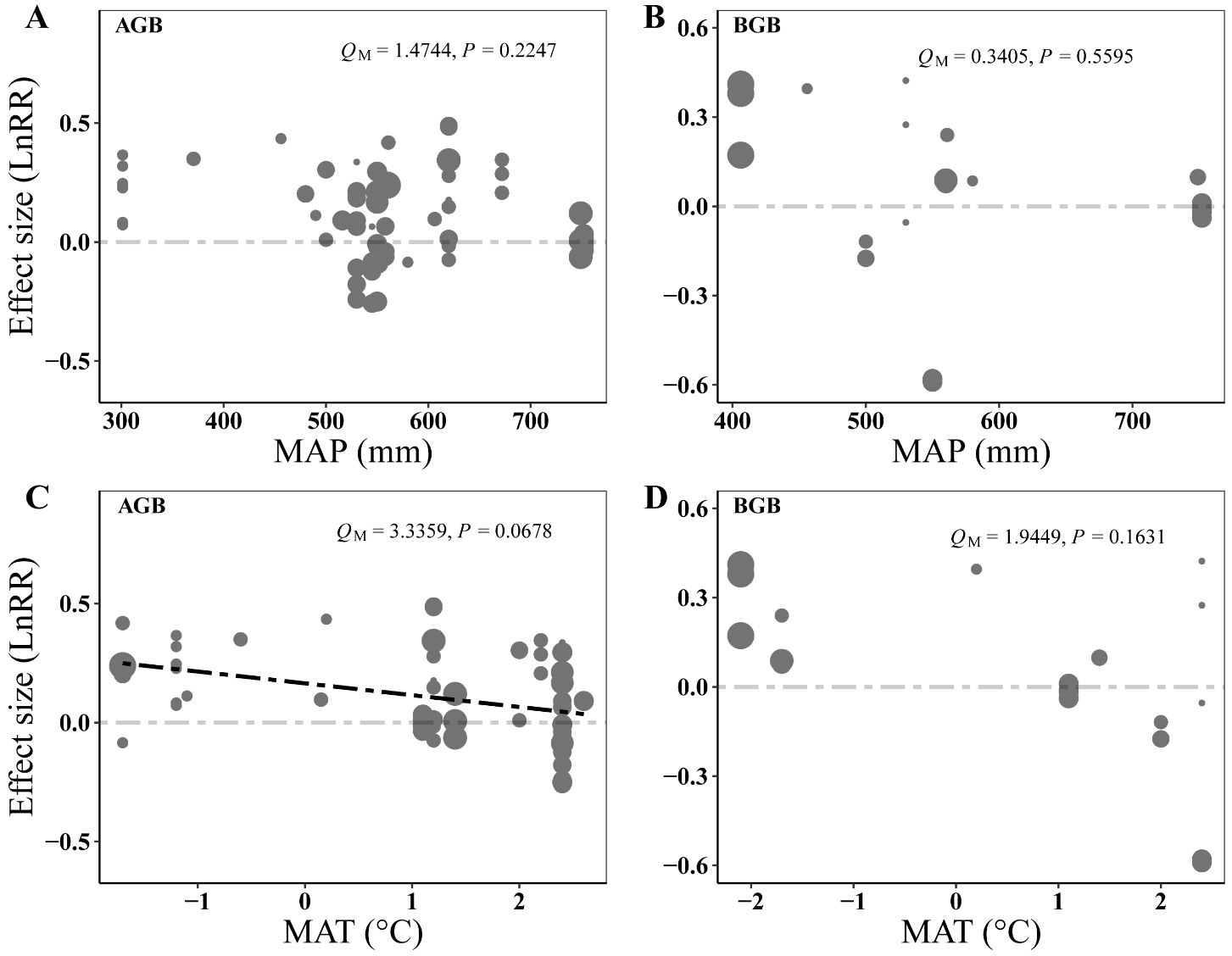


**FIGURE S2.** Results of meta-regressions relating the P addition effect on AGB (A, C) and BGB (B, D) impact (effect size, LnRR) to the MAP and MAT, respectively. The paralleled gray dashed lines represent an effect size (LnRR) of zero.


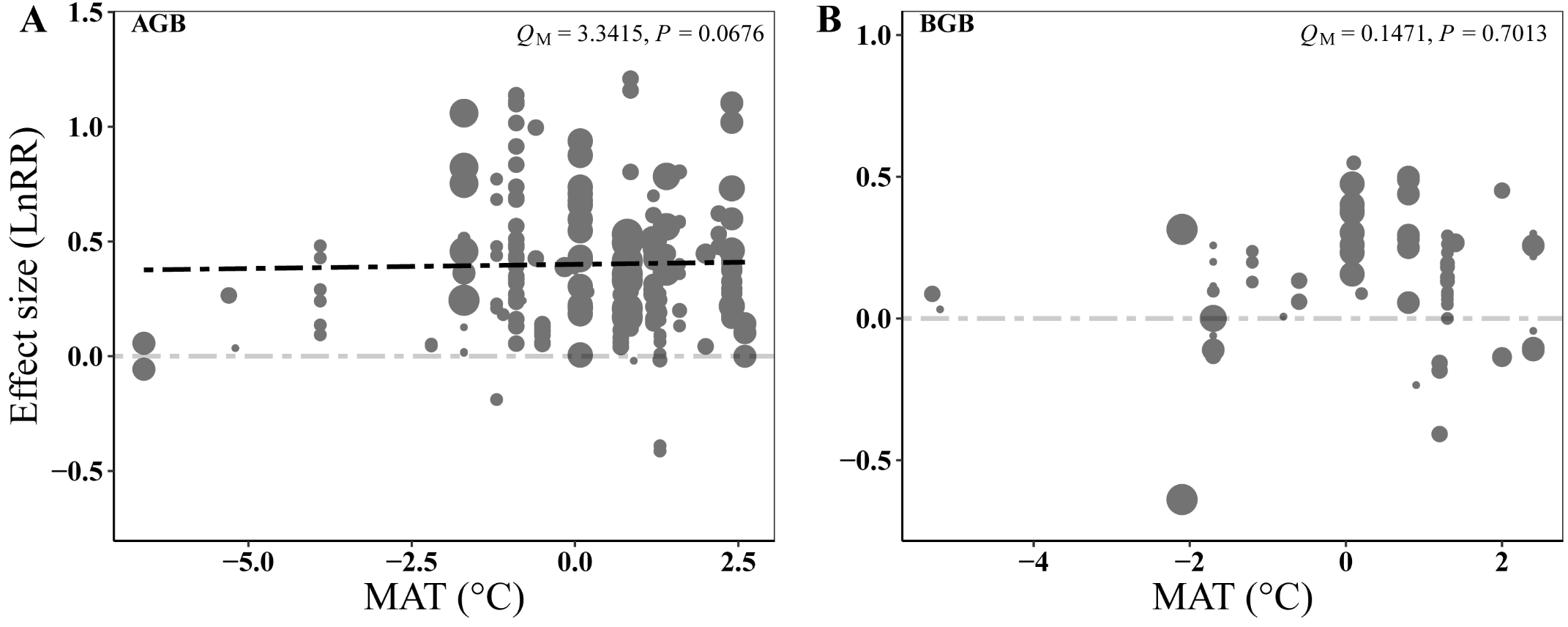


**FIGURE S3.** Results of meta-regressions relating the N addition effect on AGB (A) and BGB (B) impact (effect size, LnRR) to the MAT. The paralleled gray dashed lines represent an effect size (LnRR) of zero.

**Studies included in our meta-analysis. Chinese studies are indicated by blue font.**

[1] Wang, W., & De, K. (2015). The influences of different nitrogen types and dose rates on alpine meadow aboveground biomass and nutrients in Cheng Duo. Acta Agrestia Sinica, 23(5), 968-977.

[2] Guo, H., De, K., Lu, G., Wang, W., & Zhang, M. (2014). Effect of different fertilizer and fertilizer quantity on alpine meadow grassland in Sanjiangyuan region. Chinese Qinghai Journal of Animal and Veterinary Sciences, 44(6), 8-12.

[3] Zong, N., & Shi, P. (2020). Differential responses of community structure and production, and the sensitivities of different alpine grassland to nitrogen addition. Acta Ecologica Sinica, 40(12), 4000-4010.

[4] Jing, M., Ma, Y., Li, S., & Li, M. (2014). Effect of fertilizing nitrogen rate on degraded glassland of alpine meadow in Qilian mountain. Chinese Qinghai Journal of Animal and Veterinary Sciences, 4(44), 5-8.

[5] Chen, X., Yang, J., Gen, G., Wang, Y., Wang, D., Yang, K., & Zhou, Y. (2021). Effects of different fertilization treatments on alpine grassland. Journal of Southwest Minzu University (Natural Science Edition), 47(4), 342-347.

[6] Wang, L., Shi, J., Dong, Q., Yin, Y., Wang, X., Yu, Y., & Zhang, C. (2019). Effects of nitrogen and phosphorus addition on community diversity and biomass of alpine steppe. Acta Agrestia Sinica, 27(6), 1633-1642.

[7] Zhang, R., Shi, X., Li, W., Wang, G., & Guo, R. (2016). Effects of nitrogen and phosphorus addition on the plant aboveground biomass on a sub-alpine meadow. Ecological Science, 35(5), 15-20. doi:10.14108/j.cnki.1008-8873.2016.05.003.

[8] Xin, X., Wang, G., Yang, Y., & Ren, Z. (2014). Effects of N, P addition on above/below ground biomass allocation in a sub-alpine meadow. Ecological Science, 33(3), 452-458. doi:10.3969/j.issn.

[9] Yang, L., Liu, H., Li, C., Li, F., Xu, W., & Zhou, G. (2015). Effects of nitrogen, phosphorus and potassium fertilizer applications on plant community structure in a degraded alpine steppe. Chinese Journal of Ecology, 34(1), 25-32. doi:10.13292/j.1000-4890.2015.0005.

[10] Zhang, J., Li, Q., Ren, Z., Yang, X., & Wang, G. (2010). Effects of nitrogen addition on species richness and relationship between species richness and aboveground productivity of alpine meadow of the Qinghai-Tibetan Plateau, China. Chinese Journal of Plant Ecology, 34(10), 1125-1131. doi:10.3773/j.issn.1005-264x.2010.10.001.

[11] Li, C., Liang, D., Li, X., & Sun, H. (2020). Effect of nitrogen addition on vegetation restoration of different slope degraded alpine grassland. Journal of Qinghai University, 38(4), 1-6.

[12] Liu, X., Luo, J., Deng, D., Chen, D., Wang, L., & Zhou, J. (2018). Effects of nitrogen addition on alpine grassland ecosystems degraded to different extents. Pratacultural Science, 35(12), 2773-2783.

[13] Shen, H., Dong, S., Li, S., Xu, Y., Han, Y., & Zhang, J. (2019). Effects of nitrogen addition on the quantitative characteristics and photosynthesis of different plant functional groups in an alpine meadow of Qinghai-Tibetan Plateau. Chinese Journal of Ecology, 38(5), 1276-1284.

[14] Cao, F., Liu, R., Huang, G., Wu, H., Zhao, C., & Li, W. (2021). Effect of short-term nitrogen addition on productivity and plant diversity of subalpine grassland in Qilian Mountains. Acta Ecologica Sinica, 41(12), 5034-5044.

[15] Zhang, C. (2014). Effects of grazing and fertilization on community productivity and species richness in eastern alpine meadow of Tibetan plateau. Pratacultural Science, 31(12), 2293-2300.

[16] Shen, Z., Zhou, X., Chen, Z., & Zhou, H. (2002). Response of plant groups to simulated rainfall and nitrogen supply in alpine *Kobresia Humilis* meadow. Acta Phytoecologica Sinica, 26(3), 288-294.

[17] Sun, X., Chen, J., Li, J., Li, L., Han, G., & Chen, N. (2018). Hierarchical responses of plant stoichiometry to phosphorus addition in an alpine meadow community Chinese Journal of Plant Ecology, 42(1), 78-85. doi:10.17521/cjpe.2017.0253.

[18] Qin, S., Fang, K., Wang, G., Peng, Y., Zhang, D., Li, F., . . . Yang, Y. (2018). Responses of exchangeable base cations to continuously increasing nitrogen addition in alpine steppe: A case study of *Stipa purpurea* steppe Chinese Journal of Plant Ecology, 42(1), 95-104. doi:10.17521/cjpe.2017.0100.

[19] Li, C., Li, Q., Zhao, L., & Zhao, X. (2016). Responses of plant community biomass to nitrogen and phosphorus additions in natural and restored grasslands around Qinghai Lake Basin. Chinese Journal of Plant Ecology, 40(10), 1015-1027. doi:10.17521/cjpe.2016.0048.

[20] Zong, N., Shi, P., Zhao, G., Zheng, L., Niu, B., Zhou, T., & Hou, G. (2021). Variations of nitrogen and phosphorus limitation along the environmental gradient in alpine grasslands on the Northern Xizang Plateau. Chinese Journal of Plant Ecology, 45(5), 444-455. doi:10.17521/cjpe.2020.0135.

[21] Wang, H., Niu, J., Zhang, H., & Chen, Z. (2008). Research on characteristics of plant niche and effects of fertilization on plant productivity in a Maqu alpine desertified meadow. Acta Prataculturae Sinica, 17(16), 18-24.

[22] Yao, H., Lu, J., Cai, L., Dong, B., & Zhang, R. (2009). Response of the degraded grassland vegetation characteristics to different fertilizer treatments at Maqu County. Journal of Gansu Agricultural University, 44(4), 127-131.

[23] Zong, N., Shi, P., Song, M., & Lin, L. (2012). Clipping alters the response of biomass allocation pattern under nitrogen addition in an alpine meadow on the Tibetan plateau. Journal of Natural Resources, 27(10), 1696-1707.

[24] Zhang, L., Wang, X., Zhu, Z., & Li, Y. (2018). The effects of simulated grazing intensity and fertilizing on the community characteristics and diversity in alpine meadow of Qinghai-Tibet Plateau. Ecology and Environmental Sciences, 27(3), 406-415. doi:10.16258/j.cnki.1674-5906.2018.03.002.

[25] Ren, Q., Luo, Y., Wang, H., & Liu, J. (2004). Restoration of degraded typical alpine meadowland on the Qinghai-Tibetan plateau. Acta Prataculturae Sinica, 13(2), 43-49.

[26] Liu, Y., Tang, J., Liu, P., Cai, X., & Hao, L. (2017). Responses of soil organic carbon and nitrogen to fertilization in alpine meadows on the Qinghai-Tibet Plateau. Agricultural Science-Technology and Information (10), 94-97. doi:10.15979/j.cnki.cn62-1057/s.2017.10.048.

[27] Yang, X., Ren, F., Zhou, H., & He, J. (2014). Responses of plant community biomass to nitrogen and phosphorus additions in an alpine meadow on the Qinghai-Xizang Plateau. Chinese Journal of Plant Ecology, 38(2), 159-166. doi:10.3724/sp.J.1258.2014.00014.

[28] Guo, Y., Xue, R., Wang, X., Guo, Z., & Shen, Y. (2016). Responding of plant community and soil nutrient in fenced mowing Qinghai-Tibetan meadow to fertilization. Journal of Southwest Minzu University (Natural Science Edition), 42(4), 383-392.

[29] Li, F., Zhou, G., Yang, L., Xu, W., Zhong, Z., & Song, W. (2014). Effects of fertilizer at later growth stage on Above-ground biomass in the alpine steppe of Tibetan plateau. Acta Agrestia Sinica, 22(5), 998-1006.

[30] Cao, W., Li, W., Li, X., Xu, C., Shi, S., & Han, T. (2015). Effects of nitrogen fertilization on plant community structure and soil nutrient in alpine meadow-steppe. Journal of Desert Research, 35(3), 658-666.

[31] Duan, M., Gan, Z., Guo, J., Gao, Q., & Wan, Y. (2016). Effects of fertilization on plant diversity and productivity of alpine meadow in northern Tibet. Acta Agriculturae Boreali-occidentalis Sinica, 25(11), 1696-1703.

[32] Chen, Y., Li, Z., & Du, G. (2004). Effects of fertilization on plant diversity and economic herbage groups in alpine meadow. Acta Botanica Boreali-Occidentalia Sinica, 24(3), 424-429.

[33] Zheng, H., Chen, Z., Wang, S., & Niu, J. (2007). Effects of fertilizer on plant diversity and productivity of desertified alpine grassland at Maqu, Gansu. Acta Prataculturae Sinica, 16(5), 34-39.

[34] Yang, Z., Su, F., Miao, Y., Zhong, M., & Xiao, R. (2014). Effects of fertilization and grazing on species richness in an alpine meadow of Qinghai-Xizang Plateau. Chinese Journal of Plant Ecology, 38(10), 1074-1081. doi:10.3724/sp.J.1258.2014.00101.

[35] Yang, Z., Zhang, J., CHU, L., Li, H., & Xiao, R. (2012). Effects of fertilization and mowing on community biomass compensation in eastern alpine meadow of Tibetan Plateau. Chinese Journal of Ecology, 31(9), 2276-2282. doi:10.13292/j.1000-4890.2012.0328.

[36] Gao, B., Yuan, Z., Wang, B., Gao, H., & Zhang, R. (2014). Effects of fertilization and clipping on species diversity, productivity and their relationship in subalpine meadow. Chinese Journal of Plant Ecology, 38(5), 417-424. doi:10.3724/sp.J.1258.2014.00038.

[37] Sun, X., Chen, J., Li, J., & Chen, N. (2018). Effects of nutrient addition on ecological stoichiometric characteristics and photosynthesis of representative species in a sub-alpine meadow community. Journal of Lanzhou University (Natural Science), 54(6), 804-810. doi:10.13885/j.issn.0455-2059.2018.06.013.

[38] Zhang, Y., Le, R., Gong, X., Xu, X., & Zha, X. (2008). Effects of fertilization and mowing on productivity of alpine meadows. Prataculture & Animal Husbandry(3), 6-9.

[39] Zhang, T., Chen, X., Zhao, J., Wang, X., Zhang, R., Bai, Y., . . . Shang, Z. (2015). Effects of Carbon and Nitrogen Addition on the Plant Species Diversity and Biomass of Typical Alpine Meadow in Tibet of China. Ecology and Environmental Sciences, 24(10), 1604-1610. doi:10.16258/j.cnki.1674-5906.2015.10.003.

[40] Yang, Y., Bai, R., Li, S., Li, Z., Li, H., & Shen, Y. (2017). Responses of species abundance distribution and community similarity to nitrogen and phosphorus additions in alpine meadow. Acta Ecologica Sinica, 37(7), 2290-2299.

[41] Zong, N., Duan, C., Geng, S., Chai, X., Shi, P., & He, Y. (2018). Effects of warming and nitrogen addition on community production and biomass allocation in an alpine meadow. Chinese Journal of Applied Ecology, 29(1), 59-67.

[42] Wang, H., Zhang, L., & Zhu, Z. (2013). Effects of clipping and fertilizing on the relationships between species diversity and ecosystem functioning and mechanisms of community stability in alpine meadow. Chinese Journal of Plant Ecology, 37(4), 279-295. doi:10.3724/sp.J.1258.2013.00028.

[43] Chen, X., Wang, G., Sun, J., Zhang, T., & Mao, T. (2020). Effects of warming and N fertilization on ecosystem respiration of an alpine swamp meadow in the source area of the Yangtze River. Acta Agrestia Sinica, 28(1), 193-199.

[44] Zhou, X., Zhu, H., Li, Y., Yuan, F., & Fan, R. (2011). Community compensatory mechanism under clipping, fertilizing and watering treatment in alpine meadow. Journal of Lanzhou University (Natural Science), 47(3), 50-57.

[45] Li, X., Zhu, Z., Zhou, X., Yuan, F., Fan, R., & Xu, M. (2011). Effects of clipping, fertilizing and watering on the relationship between species diversity, functional diversity and primary productivity in alpine meadow of China. Chinese Journal of Plant Ecology, 35(11), 1136-1147. doi:10.3724/sp.J.1258.2011.01136.

[46] Pan, S., Kong, B., Yao, T., Wei, X., Li, Y., & Zhu, H. (2015). Effects of clipping and fertilizing on the relationship between functional diversity and aboveground net primary productivity in an alpine meadow. Chinese Journal of Plant Ecology, 39(9), 867-877. doi:10.17521/cjpe.2015.0083.

[47] Zhang, H. (2017). Effects of land use types and fertilization on soil organic carbon storages in alpine meadows. Lanzhou University.

[48] Luo, Y. (2020). Effects of nitrogen and phosphorus addtion on belowground net primary productivity of Kobresia pygmaea meadow. Beijing Forestry University.

[49] Han, F. (2014). Effect of nitrogen and phosphorus addition on species diversity and plant height in the sub-alpine meadow. Lanzhou University.

[50] Fu, Y. (2020). Responses and mechanisms of ecosystem carbon fluxes to multiple nutrient addition and drought in an alpine meadow. Tibet University.

[51] Liu, S. (2021). Adaptation mechanism of alpine meadow plant community and stability to long-term nitrogen addtion. Southwest Minzu University.

[52] Bin, Z. (2014). Effects of silicon, nitrogen and phosphorus addition on plant community structure and productivity of alpine meadow on Qinghai-Tibetan plateau. Lanzhou University.

[53] Ji, Y. (2017). Effects of altered precipitation, nitrogen addition and warming on plant community structure in an alpine meadow on the Tibetan plateau. Nanjing Agricultural University.

[54] Tang, L. (2020). Effects of phosphorus addition on plant root characteristics and leaf traits in an alpine meadow of the northwestern Sichuan, China. Southwest Minzu University.

[55] Qi, R. (2013). Response of plant community to nitrogen and phosphorus additions in sub-alpine meadow of the Qinghai-Tibetan plateau. Lanzhou University.

[56] Wei, X. (2011). Effects of fertilization on vegetation and soil of degraded grassland on northern Tibetan plateau. Tibet University.

[57] Xiao, R. (2012). Effects of long-term fertilization on the community structure diversity of prokaryotic microbes and ammonia oxidizers in the alpine meadow soil on Tibet plateau. Lanzhou University.

[58] Yao, H. (2008). Effects of fertilizer application on degenerative meadow community characteristics at Maqu. Gansu Agricultural University.

[59] Li, B. (2016). Effect of fertilization on GRSP and environmental factors in alpine meadows on the Qinghai-Tibetan plateau. Lanzhou University.

[60] Dong, J., Cui, X., Wang, S., Wang, F., Pang, Z., Xu, N., . . . Wang, S. (2016). Changes in biomass and quality of alpine steppe in response to N & P fertilization in the Tibetan plateau. PLoS One, 11(5). doi:10.1371/journal.pone.0156146.

[61] Zhou, X., Guo, Z., Zhang, P., Li, H., Chu, C., Li, X., & Du, G. (2017). Different categories of biodiversity explain productivity variation after fertilization in a Tibetan alpine meadow community. Ecol Evol, 7(10), 3464-3474. doi:10.1002/ece3.2723.

[62] Li, J. H., Yang, Y. J., Li, B. W., Li, W. J., Wang, G., & Knops, J. M. (2014). Effects of nitrogen and phosphorus fertilization on soil carbon fractions in alpine meadows on the Qinghai-Tibetan plateau. PLoS One, 9(7). doi:10.1371/journal.pone.0103266.

[63] Niu, K., Choler, P., de Bello, F., Mirotchnick, N., Du, G., & Sun, S. (2014). Fertilization decreases species diversity but increases functional diversity: A three-year experiment in a Tibetan alpine meadow. Agriculture, Ecosystems & Environment, 182, 106-112. doi:10.1016/j.agee.2013.07.015.

[64] Li, J., Zhang, C., Yang, Z., Guo, H., Zhou, X., & Du, G. (2017). Grazing and fertilization influence plant species richness via direct and indirect pathways in an alpine meadow of the eastern Tibetan plateau. Grass and Forage Science, 72(2), 343-354. doi:10.1111/gfs.12232.

[65] Xu, D., Fang, X., Zhang, R., Gao, T., Bu, H., & Du, G. (2015). Influences of nitrogen, phosphorus and silicon addition on plant productivity and species richness in an alpine meadow. AoB Plants, 7. doi:10.1093/aobpla/plv125.

[66] Jing, X., Yang, X., Ren, F., Zhou, H., Zhu, B., & He, J.-S. (2016). Neutral effect of nitrogen addition and negative effect of phosphorus addition on topsoil extracellular enzymatic activities in an alpine grassland ecosystem. Applied Soil Ecology, 107, 205-213. doi:10.1016/j.apsoil.2016.06.004.

[67] Li, W., Cheng, J. M., Yu, K. L., Epstein, H. E., & Du, G. Z. (2015). Niche and neutral processes together determine diversity loss in response to fertilization in an alpine meadow community. PLoS One, 10(8). doi:10.1371/journal.pone.0134560.

[68] Li, W., Cheng, J. M., Yu, K. L., Epstein, H. E., Guo, L., Jing, G. H., . . . Du, G. Z. (2015). Plant functional diversity can be independent of species diversity: observations based on the impact of 4-Yrs of nitrogen and phosphorus additions in an alpine meadow. PLoS One, 10(8), e0136040. doi:10.1371/journal.pone.0136040.

[69] Li, C., Zheng, Z., Peng, Y., Nie, X., Yang, L., Xiao, Y., & Zhou, G. (2019). Precipitation and nitrogen addition enhance biomass allocation to aboveground in an alpine steppe. Ecology and Evolution, 9(21), 12193-12201. doi:10.1002/ece3.5706.

[60] Song, M. H., & Yu, F. H. (2015). Reduced compensatory effects explain the nitrogen-mediated reduction in stability of an alpine meadow on the Tibetan Plateau. New Phytologist, 207(1), 70-77. doi:10.1111/nph.13329.

[71] Jiang, J., Zong, N., Song, M., Shi, P., Ma, W., Fu, G., . . . Ouyang, H. (2013). Responses of ecosystem respiration and its components to fertilization in an alpine meadow on the Tibetan plateau. European Journal of Soil Biology, 56, 101-106. doi:10.1016/j.ejsobi.2013.03.001.

[72] Li, W., Cheng, J., Yu, K., Epstein, H. E., & Du, G. (2015). Short-term responses of an alpine meadow community to removal of a dominant species along a fertilization gradient. Journal of Plant Ecology, 8(5), 513-522. doi:10.1093/jpe/rtu039.

[73] Sun, X., Yu, K., Shugart, H. H., & Wang, G. (2015). Species richness loss after nutrient addition as affected by N:C ratios and phytohormone GA3 contents in an alpine meadow community. Journal of Plant Ecology, 9(2), 201-211.

[74] Yang, Z., Guo, H., Zhang, J., & Du, G. (2013). Stochastic and deterministic processes together determine alpine meadow plant community composition on the Tibetan plateau. Oecologia, 171(2), 495-504. doi:10.1007/s00442-012-2433-6.

[75] Zhou, X., Wang, Y., Zhang, P., Guo, Z., Chu, C., & Du, G. (2015). The effects of fertilization on the trait–abundance relationships in a Tibetan alpine meadow community. Journal of Plant Ecology, 9(2), 144-152.

[76] Yang, Z., van Ruijven, J., & Du, G. (2011). The effects of long-term fertilization on the temporal stability of alpine meadow communities. Plant and Soil, 345(1-2), 315-324. doi:10.1007/s11104-011-0784-0.

[77] Wang, C., Wang, G., Wang, Y., Rafique, R., Ma, L., Hu, L., & Luo, Y. (2015). Urea addition and litter manipulation alter plant community and soil microbial community composition in a *Kobresia humilis* meadow. European Journal of Soil Biology, 70, 7-14. doi:10.1016/j.ejsobi.2015.06.003.

[78] Ren, Z., Li, Q., Chu, C., Zhao, L., Zhang, J., Dexiecuo, A., . . . Wang, G. (2009). Effects of resource additions on species richness and ANPP in an alpine meadow community. Journal of Plant Ecology, 3(1), 25-31. doi:10.1093/jpe/rtp034.

[79] Wang, F., Shi, G., Nicholas, O., Yao, B., Ji, M., Wang, W., . . . Lee, J. (2018). Ecosystem nitrogen retention is regulated by plant community trait interactions with nutrient status in an alpine meadow. Journal of Ecology, 106(4), 1570-1581. doi:10.1111/1365-2745.12924.

[80] Ren, F., Song, W., Chen, L., Mi, Z., Zhang, Z., Zhu, W., . . . He, J.-S. (2016). Phosphorus does not alleviate the negative effect of nitrogen enrichment on legume performance in an alpine grassland. Journal of Plant Ecology, 10(5), 822-830. doi:10.1093/jpe/rtw089.

[81] He, D., Xiang, X., He, J.-S., Wang, C., Cao, G., Adams, J., & Chu, H. (2016). Composition of the soil fungal community is more sensitive to phosphorus than nitrogen addition in the alpine meadow on the Qinghai-Tibetan Plateau. Biology and Fertility of Soils, 52(8), 1059-1072. doi:10.1007/s00374-016-1142-4.

[82] Zong, N., & Shi, P. (2019). Enhanced community production rather than structure improvement under nitrogen and phosphorus addition in severely degraded alpine meadows. Sustainability, 11(7). doi:10.3390/su11072023.

[83] Xiang, X., He, D., He, J.-S., Myrold, D. D., & Chu, H. (2017). Ammonia-oxidizing bacteria rather than archaea respond to short-term urea amendment in an alpine grassland. Soil Biology and Biochemistry, 107, 218-225. doi:10.1016/j.soilbio.2017.01.012.

[84] Kwaku, E. A., Dong, S., Shen, H., Li, W., Sha, W., Su, X., . . . Zhao, Z. (2021). Biomass and Species Diversity of Different Alpine Plant Communities Respond Differently to Nitrogen Deposition and Experimental Warming. Plants (Basel), 10(12). doi:10.3390/plants10122719.

[85] Jiang, J., Shi, P., Zong, N., Fu, G., Shen, Z., Zhang, X., & Song, M. (2014). Climatic patterns modulate ecosystem and soil respiration responses to fertilization in an alpine meadow on the Tibetan plateau, China. Ecological Research, 30(1), 3-13. doi:10.1007/s11284-014-1199-1.

[86] Zhang, C., Willis, C. G., Ma, Z., Ma, M., Csontos, P., Baskin, C. C., . . . Du, G. (2019). Direct and indirect effects of long-term fertilization on the stability of the persistent seed bank. Plant and Soil, 438(1-2), 239-250. doi:10.1007/s11104-019-04024-x.

[87] Chen, W., Zhou, H., Wu, Y., Wang, J., Zhao, Z., Li, Y., . . . Xue, S. (2020). Direct and indirect influences of long-term fertilization on microbial carbon and nitrogen cycles in an alpine grassland. Soil Biology and Biochemistry, 149. doi:10.1016/j.soilbio.2020.107922.

[88] Liu, W., Jiang, Y., Wang, G., Su, Y., Smoak, J. M., Liu, M., & Duan, B. (2021). Effects of N addition and clipping on above and belowground plant biomass, soil microbial community structure, and function in an alpine meadow on the Qinghai-Tibetan Plateau. European Journal of Soil Biology, 106. doi:10.1016/j.ejsobi.2021.103344.

[89] Mu, Z., Dong, S., Li, Y., Li, S., Shen, H., Zhang, J., . . . Zhao, Z. (2021). Soil bacterial community responses to N application and warming in a Qinghai-Tibetan plateau alpine steppe. Frontiers in Ecology and Evolution, 9. doi:10.3389/fevo.2021.709518.

[90] Song, B., Sun, J., Zhou, Q., Zong, N., Li, L., & Niu, S. (2017). Initial shifts in nitrogen impact on ecosystem carbon fluxes in an alpine meadow: patterns and causes. Biogeosciences, 14(17), 3947-3956. doi:10.5194/bg-14-3947-2017.

[91] Zhang, L., Zhu, T., Liu, X., Nie, M., Xu, X., & Zhou, S. (2020). Limited inorganic N niche partitioning by nine alpine plant species after long-term nitrogen addition. Science of Total Environment, 718. doi:10.1016/j.scitotenv.2020.137270.

[92] Fang, H., Cheng, S., Yu, G., Cooch, J., Wang, Y., Xu, M., . . . Li, Y. (2014). Low-level nitrogen deposition significantly inhibits methane uptake from an alpine meadow soil on the Qinghai–Tibetan plateau. Geoderma, 213, 444-452. doi:10.1016/j.geoderma.2013.08.006.

[93] Li, J. H., Li, F., Li, W. J., Chen, S., Abbott, L. K., & Knops, J. M. H. (2018). Nitrogen additions promote decomposition of soil organic carbon in a Tibetan alpine meadow. Soil Science Society of America Journal, 82(3), 614-621. doi:10.2136/sssaj2017.12.0417.

[94] Luo, R., Fan, J., Wang, W., Luo, J., Kuzyakov, Y., He, J. S., . . . Ding, W. (2019). Nitrogen and phosphorus enrichment accelerates soil organic carbon loss in alpine grassland on the Qinghai-Tibetan plateau. Science of Total Environment, 650(1), 303-312. doi:10.1016/j.scitotenv.2018.09.038.

[95] Zong, N., Shi, P., Song, M., Zhang, X., Jiang, J., & Chai, X. (2016). Nitrogen critical loads for an alpine meadow ecosystem on the Tibetan plateau. Environmental Management, 57(3), 531-542. doi:10.1007/s00267-015-0626-6.

[96] Yan, Y., Ganjurjav, H., Hu, G., Liang, Y., Li, Y., He, S., . . . Gao, Q. (2018). Nitrogen deposition induced significant increase of N2O emissions in an dry alpine meadow on the central Qinghai–Tibetan plateau. Agriculture, Ecosystems & Environment, 265, 45-53. doi:10.1016/j.agee.2018.05.031.

[97] Yang, Y., Xiao, Y., Li, C., Wang, B., Gao, Y., & Zhou, G. (2021). Nitrogen addition, rather than altered precipitation, stimulates nitrous oxide emissions in an alpine steppe. Ecology and Evolution, 11(21), 15153-15163. doi:10.1002/ece3.8196.

[98] Peng, Y., Li, F., Zhou, G., Fang, K., Zhang, D., Li, C., . . . Yang, Y. (2017). Nonlinear response of soil respiration to increasing nitrogen additions in a Tibetan alpine steppe. Environmental Research Letters, 12(2). doi:10.1088/1748-9326/aa5ba6.

[99] Xu, X., Wanek, W., Zhou, C., Richter, A., Song, M., Cao, G., . . . Kuzyakov, Y. (2014). Nutrient limitation of alpine plants: Implications from leaf N : P stoichiometry and leaf δ^15^N. Journal of Plant Nutrition and Soil Science, 177(3), 378-387. doi:10.1002/jpln.201200061.

[100] Gao, Y., Cooper, D. J., & Ma, X. (2016). Phosphorus additions have no impact on plant biomass or soil nitrogen in an alpine meadow on the Qinghai-Tibetan plateau, China. Applied Soil Ecology, 106, 18-23. doi:10.1016/j.apsoil.2016.04.020.

[101] Zong, N., Shi, P., Jiang, J., Song, M., Xiong, D., Ma, W., . . . Shen, Z. (2013). Responses of ecosystem CO_2_ fluxes to short-term experimental warming and nitrogen enrichment in an alpine meadow, northern Tibet plateau. The Scientific World Journal. doi:10.1155/2013/415318.

[102] Zong, N., Song, M., Shi, P., Jiang, J., Zhang, X., & Shen, Z. (2014). Timing patterns of nitrogen application alter plant production and CO_2_ efflux in an alpine meadow on the Tibetan plateau, China. Pedobiologia, 57(4-6), 263-269. doi:10.1016/j.pedobi.2014.08.001.

[103] Zong, N., Shi, P., Zheng, L., Zhou, T., Cong, N., Hou, G., . . . Zhu, J. (2021). Restoration effects of fertilization and grazing exclusion on different degraded alpine grasslands: Evidence from a 10-year experiment. Ecological Engineering, 170. doi:10.1016/j.ecoleng.2021.106361.

[104] Bai, W., Wang, G., Xi, J., Liu, Y., & Yin, P. (2019). Short-term responses of ecosystem respiration to warming and nitrogen addition in an alpine swamp meadow. European Journal of Soil Biology, 92, 16-23. doi:10.1016/j.ejsobi.2019.04.003.

[105] Xiao, Y., Li, C., Yang, Y., Peng, Y., Yang, Y., & Zhou, G. (2020). Soil fungal community composition, not assembly process, was altered by nitrogen addition and precipitation changes at an alpine steppe. Frontiers in Microbiology, 11, 579072. doi:10.3389/fmicb.2020.579072.

[106] Wei, D., Xu, R., Liu, Y., Wang, Y., & Wang, Y. (2014). Three-year study of CO_2_ efflux and CH_4_/N_2_O fluxes at an alpine steppe site on the central Tibetan plateau and their responses to simulated N deposition. Geoderma, 232-234, 88-96. doi:10.1016/j.geoderma.2014.05.002.

[107] Yan, X. (2021) Mechanisms and interaction effects of short-term nitrogen and phosphorus fertilization and cold-season grazing on plant diversity and productivity of alpine meadow. Lanzhou University.
